# Serum hypocretin, neurofilament heavy chain, and interleukin-1β as combined predictors of sleep disorders following acute ischemic stroke

**DOI:** 10.3389/fnins.2026.1814307

**Published:** 2026-05-07

**Authors:** Chaowei Wang, Yunqing Mao, Shuo Wang, Li Liu, Guang Yao

**Affiliations:** 1 Department of Neurology, The First Affiliated Hospital of Henan Medical University, Henan Provincial Key Laboratory of Neural Repair and Protein Modification, Henan Provincial International Joint Laboratory of Neural Repair for Senile Dementia, Henan Provincial Engineering Research Center of Neural Repair, Henan Provincial Engineering Technology Research Center of Neural Repair, and Henan Provincial Medical Key Laboratory of Neurology, Weihui, Henan, China; 2 Department of Neuroelectrophysiology, The First Affiliated Hospital of Henan Medical University, Henan Provincial Key Laboratory of Neural Repair and Protein Modification, Henan Provincial International Joint Laboratory of Neural Repair for Senile Dementia, Henan Provincial Engineering Research Center of Neural Repair, Henan Provincial Engineering Technology Research Center of Neural Repair, and Henan Provincial Medical Key Laboratory of Neurology, Weihui, Henan, China; 3 School of Mathematics and Statistics, Xinxiang University, Xinxiang, Henan, China

**Keywords:** acute ischemic stroke, hypocretin, interleukin-1β, neurofilament heavy chain, sleep disorders

## Abstract

**Background:**

Sleep disorders represent a common and impactful complication following acute ischemic stroke (AIS). This study aimed to identify clinical risk factors and evaluate the predictive value of serum hypocretin (Hcrt), neurofilament heavy chain (NfH), and interleukin-1 beta (IL-1β) for post-stroke sleep disorders.

**Methods:**

We conducted a retrospective observational study of 256 patients with AIS. Patients were classified into sleep disorder (*n* = 161) and non-sleep disorder (*n* = 95) groups based on their Pittsburgh Sleep Quality Index scores 7 days after stroke onset. Fasting serum levels of Hcrt, NfH, and IL-1β were measured upon admission. We utilized multivariate logistic regression and receiver operating characteristic (ROC) curves to evaluate predictive performance. The combined model was internally validated using 1,000 bootstrap resamples to assess optimism-corrected discriminative performance.

**Results:**

Sleep disorders were present in 62.9% of patients. Nine independent risk factors were identified: age ≥ 65 years (OR = 2.059), snoring history (OR = 1.980), prior stroke (OR = 2.036), lower ADL scores (OR = 1.839), higher HAMD (OR = 1.726) and NIHSS scores (OR = 1.677), decreased serum Hcrt (OR = 1.863), elevated NfH (OR = 2.020), and elevated IL-1β (OR = 1.793; all *p* < 0.05). Individual biomarker AUCs ranged from 0.742 to 0.781, whereas the combined three-biomarker model achieved a significantly superior AUC of 0.874 (sensitivity 88.82%, specificity 71.58%). Bootstrap internal validation yielded a mean optimism-corrected AUC of 0.861 (95% CI: 0.812–0.903), indicating robust model performance with minimal overfitting.

**Conclusion:**

Clinical variables alongside altered levels of Hcrt, NfH, and IL-1β serve as independent predictors of post-stroke sleep disorders. The combined three-biomarker panel, reflecting neuroendocrine dysregulation, axonal injury, and systemic inflammation, demonstrates substantially superior predictive accuracy over individual biomarkers and offers a clinically practical tool for early identification of high-risk patients.

## 1 Introduction

Acute ischemic stroke (AIS) is one of the most prevalent neurological emergencies, resulting from acute cerebrovascular occlusion and subsequent focal cerebral ischemia. Beyond the well-recognized motor and cognitive sequelae, sleep disorders represent a common yet often underappreciated non-motor complication following AIS, with reported incidence rates ranging from 50 to 70% (Kojic et al., 2022). These disturbances encompass a broad spectrum of manifestations, including insomnia, excessive daytime sleepiness, sleep fragmentation, and alterations in sleep architecture such as reduced slow-wave sleep (Baillieul et al., 2023). Accumulating evidence indicates that post-stroke sleep disorders are associated with delayed neurological recovery, increased risk of cognitive impairment, and a higher incidence of emotional disturbances (Zhang P. et al., 2025; Wu et al., 2024). Consequently, elucidating the mechanisms underlying sleep disorders after AIS and identifying modifiable risk factors have become important priorities in stroke rehabilitation.

The pathogenesis of post-stroke sleep disorders is considered multifactorial, involving the interplay of biological, psychological, and social factors (Denis et al., 2024; Zhang Z. et al., 2023). While conventional risk factors, such as advanced age, stroke lesion location, and pre-existing comorbidities, have been well characterized, they do not fully account for the pathophysiological complexity of this condition. In recent years, increasing attention has been directed toward neurobiochemical and neuroimmune alterations following stroke, which may constitute key biological mechanisms linking brain injury to sleep disruption.

Hypocretin (Hcrt), also known as orexin, is a neuropeptide produced by neurons in the lateral hypothalamus that plays a central role in maintaining wakefulness and regulating the sleep–wake cycle. Cerebral ischemia may compromise hypothalamic neuronal function and disrupt sleep–wake homeostasis, potentially through inflammatory cascades (Li et al., 2022). Neurofilament heavy chain (NfH), a structural protein of neuronal axons, is released into the circulation upon axonal injury and serves as a biomarker of neuroaxonal damage. Elevated NfH levels may reflect injury to neural pathways involved in sleep–wake regulation, including brainstem and thalamic circuits (Qiao et al., 2025). Interleukin-1β (IL-1β), a key proinflammatory cytokine, is released in substantial quantities following cerebral ischemia and has been implicated in the modulation of sleep architecture, particularly through its effects on slow-wave sleep (Catana et al., 2023).

Despite the growing recognition of these individual pathways, studies that simultaneously examine the contributions of neuroendocrine regulation, axonal injury, and inflammation to post-stroke sleep disorders remain limited. The present study aimed to identify the independent risk factors for sleep disorders in patients with AIS and to evaluate the individual and combined predictive value of serum Hcrt, NfH, and IL-1β levels, with the goal of providing an evidence base for the early identification of high-risk patients and the development of targeted sleep intervention strategies.

## 2 Methods

### 2.1 Study design

This was a single-center, retrospective observational study conducted at the Department of Neurology, the First Affiliated Hospital of Xinxiang Medical University, Xinxiang, Henan Province, China. The study employed a cross-sectional analytic design to identify risk factors for sleep disorders in patients with AIS and to evaluate the predictive value of serum Hcrt, NfH, and IL-1β levels for post-stroke sleep disturbances. No interventions were administered; all data were derived from routine clinical assessments and standard-of-care laboratory investigations.

This study was approved by the Ethics Committee of the First Affiliated Hospital of Xinxiang Medical University. The study was conducted in accordance with the principles of the Declaration of Helsinki. Given the retrospective observational nature of the study and the use of de-identified clinical data, the requirement for individual written informed consent was waived by the institutional ethics committee. All patient data were anonymized prior to analysis to protect participant confidentiality.

### 2.2 Participants

#### 2.2.1 Study population and recruitment

**Figure 1:**
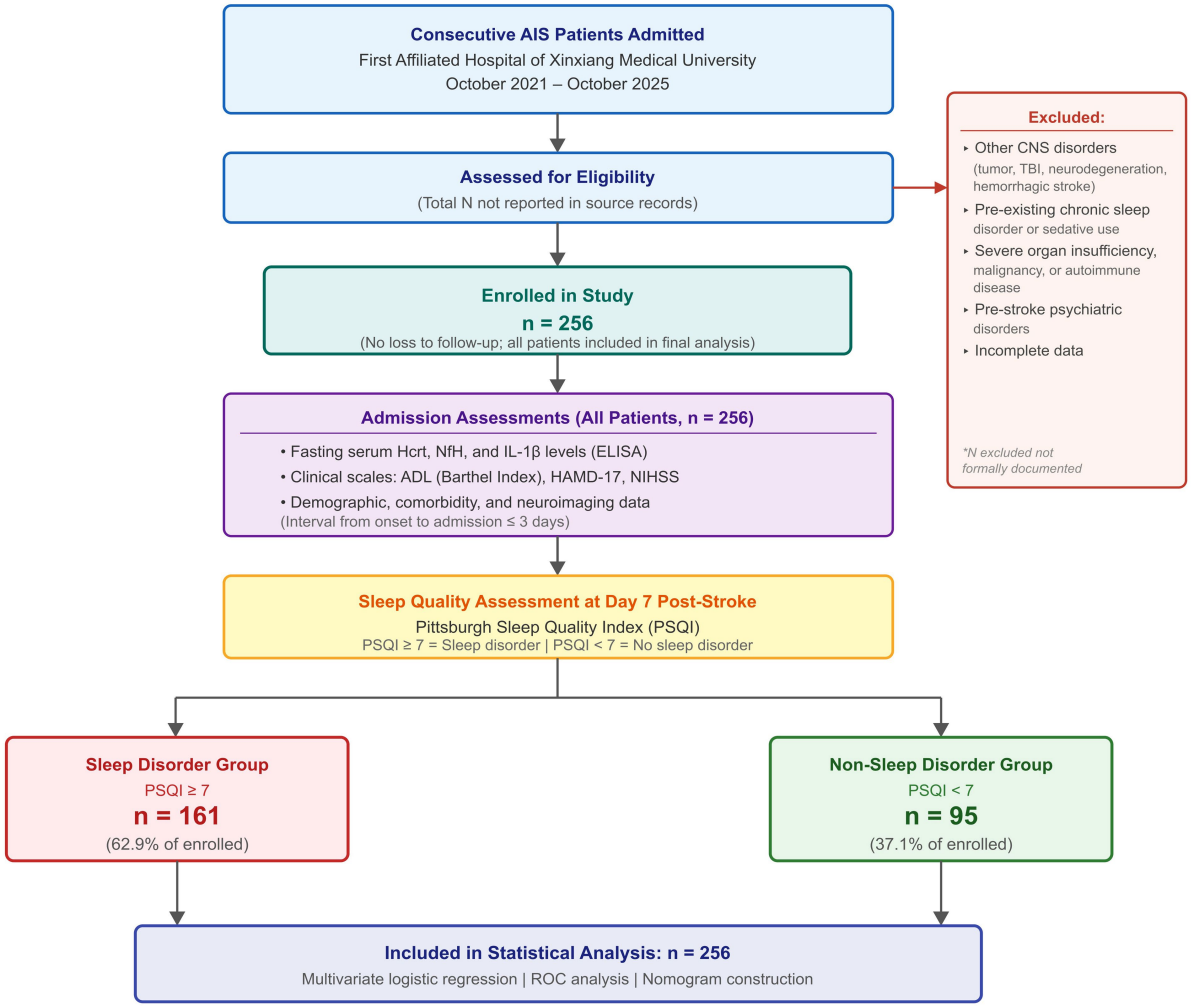
STROBE-compliant study enrollment and patient flow diagram. AIS, acute ischemic stroke; PSQI, Pittsburgh Sleep Quality Index; Hcrt, hypocretin; NfH, neurofilament heavy chain; IL-1β, interleukin-1β; ADL, activities of daily living; HAMD, Hamilton Depression Rating Scale; NIHSS, National Institutes of Health Stroke Scale; ELISA, enzyme-linked immunosorbent assay. *Total number of patients screened prior to eligibility assessment was not formally recorded in the electronic medical record system.

A total of 256 consecutive patients diagnosed with AIS who were admitted to the First Affiliated Hospital of Xinxiang Medical University between October 2021 and October 2025 were enrolled. The study enrollment and patient flow are illustrated in Figure 1. Patients were identified at the time of admission and their clinical data were collected from the electronic medical record system. Patients were classified into two groups on the basis of sleep quality assessed 7 days after stroke onset using the Pittsburgh Sleep Quality Index (PSQI): the sleep disorder group (PSQI score ≥ 7; *n* = 161) and the non-sleep disorder group (PSQI score < 7; *n* = 95) (Shen et al., 2021).

#### 2.2.2 Inclusion criteria

1. Diagnosis of AIS confirmed by neuroimaging (cranial computed tomography and/or magnetic resonance imaging), in accordance with the Chinese Guidelines for the Diagnosis and Treatment of Acute Ischemic Stroke 2018 (Liu et al., 2023; Sun D. et al., 2025);
2. AIS confirmed as the primary presenting neurological event during the study enrollment period, regardless of prior stroke history; age ≥ 18 years;
3. Interval from symptom onset to hospital admission ≤ 3 days;
4. Complete clinical, laboratory, and follow-up data available for analysis.

#### 2.2.3 Exclusion criteria

1. History of other central nervous system disorders, including brain tumors, traumatic brain injury, neurodegenerative diseases, or hemorrhagic stroke;
2. Pre-existing chronic sleep disorders diagnosed prior to stroke onset, including chronic insomnia disorder, restless legs syndrome, or obstructive sleep apnea syndrome, or chronic use of sedative-hypnotic medications;
3. Severe cardiac, hepatic, or renal insufficiency; advanced malignancy; or active autoimmune disease;
4. Comorbid psychiatric disorders including major depressive disorder, schizophrenia, or bipolar disorder diagnosed prior to the index stroke.

### 2.3 Data collection and clinical assessments

Clinical data were collected by trained investigators from the First Affiliated Hospital of Xinxiang Medical University using a standardized case report form. All investigators underwent unified training on data abstraction procedures and instrument administration prior to study commencement to ensure consistency. Demographic and clinical variables recorded included age, sex, body mass index (BMI), history of alcohol consumption, smoking history, educational attainment, history of snoring, comorbid diabetes mellitus, hyperlipidemia, hypertension, previous stroke history, and lesion location as determined by neuroimaging.

#### 2.3.1 Clinical scales

Three validated clinical instruments were administered at the time of admission. The Activities of Daily Living (ADL) scale (Barthel Index) was used to evaluate functional independence, with total scores ranging from 0 to 100, where higher scores indicate greater independence in daily living activities (Mahoney and Barthel, 1965). The Hamilton Depression Rating Scale (HAMD, 17-item version) was employed to assess depressive symptomatology, with total scores ranging from 0 to 54; a score ≥ 7 was considered indicative of depressive symptoms, and higher scores reflect greater severity (Zhou et al., 2023). Neurological deficit severity was quantified using the National Institutes of Health Stroke Scale (NIHSS), with total scores ranging from 0 to 42, where higher scores denote more severe neurological impairment (You et al., 2024).

#### 2.3.2 Sleep quality assessment

Sleep quality was assessed on day 7 after stroke onset using the Pittsburgh Sleep Quality Index (PSQI), a validated self-report questionnaire that evaluates seven domains of sleep quality. The PSQI yields a global score ranging from 0 to 21, with a score ≥ 7 serving as the threshold for clinically significant sleep disturbance. Patients who were unable to complete the PSQI independently due to aphasia or cognitive impairment were assisted by trained clinical staff with input from their caregivers.

### 2.4 Laboratory measurements

Venous blood samples were obtained from each patient at the time of the first biochemical examination after admission. Fasting blood samples were drawn into standard serum separator tubes, allowed to clot at room temperature for 30 min, and centrifuged at 3,000 rpm for 10 min. The resulting serum was separated and analyzed on the same day. Serum levels of Hcrt (μg/L), NfH (ng/L), and IL-1β (pg/mL) were measured using a ZS-210 automated biochemical analyzer (Zhongsheng Medical Technology Co., Ltd., Suzhou, China). Assays were performed using enzyme-linked immunosorbent assay (ELISA) kits according to the manufacturer’s instructions. Intra-assay and inter-assay coefficients of variation were maintained below 10%. All samples were analyzed in the same centralized laboratory under standardized operating procedures.

### 2.5 Quality control

Several measures were implemented to ensure data integrity. All original medical records were reviewed by uniformly trained researchers in accordance with pre-established study criteria to verify consistent application of diagnostic, inclusion, and exclusion criteria. The PSQI, NIHSS, and other key variables were subjected to random auditing, with a sampling ratio of at least 20%, independently verified by a second researcher to ensure data consistency. All serum biomarker assays were performed in the same laboratory using identical instruments and reagent batches, with implementation of standard internal quality control procedures. Dual independent data entry was performed, with logic verification rules applied to identify and flag abnormal or missing values, which were subsequently handled according to a predefined protocol.

### 2.6 Sample size consideration

Although this was a retrospective study without an *a priori* power calculation, a post-hoc assessment of model adequacy was conducted. With 161 outcome events (sleep disorder cases) and nine predictors entered into the multivariate logistic regression model, the events-per-variable (EPV) ratio was approximately 17.9—exceeding the conventionally recommended minimum EPV of 10 and the more conservative threshold of EPV ≥ 20 recommended when low-prevalence binary predictors are present (Ogundimu et al., 2016). The total sample of 256 patients was therefore considered adequate for the planned regression analysis.

### 2.7 Missing data

All 256 enrolled patients had complete records for primary clinical, laboratory, and outcome variables. No missing data imputation was required, consistent with the pre-specified inclusion criterion mandating complete data availability.

### 2.8 Statistical analysis

All statistical analyses were performed using SPSS version 26.0 (IBM Corporation, Armonk, NY, USA). The normality of continuous variables was assessed using the Shapiro–Wilk test. Continuous variables conforming to a normal distribution are expressed as mean ± standard deviation (*x̄* ± *s*), and intergroup comparisons were performed using independent-samples *t*-tests. Categorical variables are presented as frequencies and percentages [*n* (%)], and comparisons were conducted using Pearson’s chi-squared (χ^2^) test.

**Figure 2:**
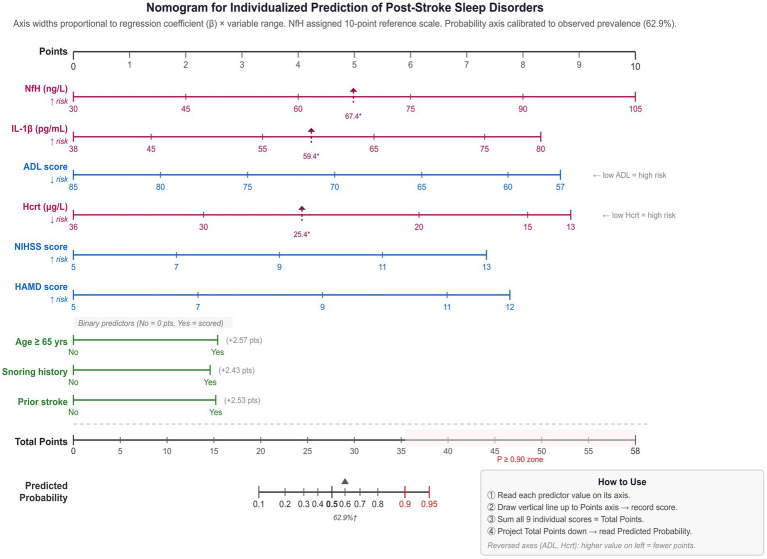
Nomogram for individualized prediction of post-stroke sleep disorders based on nine independent predictors from multivariate logistic regression. For each predictor, draw a vertical line to the Points axis; sum all scores to obtain Total Points; project downward to read the Predicted Probability. ADL and Hcrt axes are reversed (right-to-left = increasing risk). Triangles (▼) denote optimal ROC cutoffs: NfH ≥ 67.41 ng/L, IL-1β ≥ 59.41 pg/mL, Hcrt ≤ 25.42 μg/L. External validation is required before clinical use. Red, serum biomarkers; blue, continuous clinical variables; green, binary predictors. ADL, Activities of Daily Living; HAMD, Hamilton Depression Rating Scale; NIHSS, National Institutes of Health Stroke Scale; Hcrt, hypocretin; NfH, neurofilament heavy chain; IL-1β, interleukin-1β. * Optimal cutoff value from ROC/Youden index (Table 4): NfH ≥ 67.41 ng/L; IL-1β ≥ 59.41 pg/mL; Hcrt ≤ 25.42 μg/L (marked by ▲). † Probability axis calibrated from model intercept estimated at observed prevalence 62.9%; calibration requires prospective validation. Axis spans total points 20–25 (*P* = 10%–95%). Red = biomarker; Blue = clinical continuous; Green = binary.

Multivariate binary logistic regression analysis was performed to identify independent risk factors for post-stroke sleep disorders. Variables with *p* < 0.05 in the univariate analysis were entered as independent variables, with sleep disorder as the dependent variable (0 = no sleep disorder, 1 = sleep disorder). Multicollinearity among independent variables was assessed using variance inflation factors (VIF), with a threshold of VIF < 10 considered acceptable. Results are reported as regression coefficients (β), standard errors (SE), Wald χ^2^ values, odds ratios (OR), and 95% confidence intervals (CI). Model calibration was assessed using the Hosmer-Lemeshow goodness-of-fit test (a non-significant result, *p* > 0.05, indicates adequate fit). A nomogram was subsequently constructed from the nine logistic regression coefficients to facilitate individualized risk estimation. Each predictor axis was scaled proportionally to its *β* × observed variable range product, with NfH assigned the 10-point reference scale on the basis of its greatest aggregate contribution. Optimal biomarker cutoff values determined by ROC analysis were annotated on the corresponding axes, and the predicted probability axis was calibrated to the observed event prevalence of 62.9% (Figure 2).

The predictive performance of serum Hcrt, NfH, and IL-1β, individually and in combination, was evaluated using receiver operating characteristic (ROC) curve analysis. The combined predictive model was derived from the predicted probabilities of the logistic regression incorporating all three biomarkers. Areas under the ROC curve (AUC) was calculated, and pairwise AUC comparisons were performed using the DeLong test. Optimal cutoff values were determined using the Youden index (sensitivity+specificity−1). All tests were two-sided, and *p* < 0.05 was considered statistically significant.

To assess the internal validity of the combined three-biomarker predictive model and quantify any optimism in the apparent AUC, bootstrap resampling validation was performed. A total of 1,000 bootstrap samples were drawn with replacement from the original dataset. For each resample, the logistic regression model incorporating all three biomarkers was re-estimated and the AUC was recalculated. The optimism-corrected AUC was obtained by subtracting the mean optimism from the apparent AUC. A calibration plot was additionally constructed to display the concordance between predicted probabilities and observed event proportions across deciles of predicted risk (Supplementary Figure S2).

## 3 Results

### 3.1 Baseline characteristics and univariate analysis

Of the 256 patients with AIS enrolled in this study, 161 (62.9%) met the criteria for sleep disorder (PSQI ≥ 7) at 7 days after stroke onset, while 95 (37.1%) were classified into the non-sleep disorder group. This prevalence is consistent with reported incidence rates of 50–78% in the post-stroke sleep disorder literature (Kojic et al., 2022; Hasan et al., 2021).

The demographic and clinical characteristics of the two groups are summarized in Table 1. The groups were comparable with respect to BMI (23.18 ± 1.22 vs. 23.14 ± 1.17 kg/m^2^; *t* = 0.257, *p* = 0.797), sex distribution (χ^2^ = 0.045, *p* = 0.833), history of alcohol consumption (χ^2^ = 0.328, *p* = 0.567), smoking history (χ^2^ = 0.267, *p* = 0.605), educational attainment (χ^2^ = 0.363, *p* = 0.547), comorbid diabetes mellitus (χ^2^ = 0.301, *p* = 0.583), hyperlipidemia (χ^2^ = 0.056, *p* = 0.813), hypertension (χ^2^ = 0.204, *p* = 0.652), or lesion location (χ^2^ = 0.418, *p* = 0.937).

**Table 1:** Comparison of demographic, clinical, and laboratory data between the sleep disorder and non-sleep disorder groups.

Variable	Sleep disorder group (*n* = 161)	Non-sleep disorder group (*n* = 95)	t/χ^2^ value	*p* value
BMI (kg/m^2^)	23.18 ± 1.22	23.14 ± 1.17	0.257	0.797
Age, *n (%)*			7.373	0.007
< 65 years	49 (30.43)	45 (47.37)		
≥ 65 years	112 (69.57)	50 (52.63)		
Sex, *n (%)*			0.045	0.833
Male	92 (57.14)	53 (55.79)		
Female	69 (42.86)	42 (44.21)		
Alcohol consumption, *n (%)*			0.328	0.567
Yes	53 (32.92)	28 (29.47)		
No	108 (67.08)	67 (70.53)		
Smoking history, *n (%)*			0.267	0.605
Yes	68 (42.24)	37 (38.95)		
No	93 (57.76)	58 (61.05)		
Education level, *n (%)*			0.363	0.547
High school or below	106 (65.84)	59 (62.11)		
College or above	55 (34.16)	36 (37.89)		
History of snoring, *n (%)*			10.741	0.001
Yes	83 (51.55)	29 (30.53)		
No	78 (48.45)	66 (69.47)		
Diabetes mellitus, *n (%)*			0.301	0.583
Yes	44 (27.33)	23 (24.21)		
No	117 (72.67)	72 (75.79)		
Hyperlipidemia, *n (%)*			0.056	0.813
Yes	60 (37.27)	34 (35.79)		
No	101 (62.73)	61 (64.21)		
Hypertension, *n (%)*			0.204	0.652
Yes	57 (35.40)	31 (32.63)		
No	104 (64.60)	64 (67.37)		
Previous stroke, *n (%)*			8.464	0.004
Yes	93 (57.76)	37 (38.95)		
No	68 (42.24)	58 (61.05)		
Lesion location, *n (%)*			0.418	0.937
Basal ganglia	72 (44.72)	39 (41.05)		
Cerebral cortex	40 (24.84)	25 (26.32)		
Brainstem	30 (18.63)	20 (21.05)		
Cerebellum	19 (11.80)	11 (11.58)		
ADL score at admission	69.64 ± 6.35	72.22 ± 7.45	2.942	0.004
HAMD score at admission	8.97 ± 1.62	6.43 ± 0.57	14.743	<0.001
NIHSS score at admission	9.48 ± 2.26	8.75 ± 1.48	2.811	0.005
Serum Hcrt (μg/L)	21.67 ± 4.28	27.88 ± 6.26	9.406	<0.001
Serum NfH (ng/L)	74.65 ± 21.63	53.72 ± 10.76	8.805	<0.001
Serum IL-1β (pg/mL)	62.75 ± 10.33	52.34 ± 8.57	8.282	<0.001

Continuous variables are presented as mean ± SD; categorical variables as n (%). BMI, body mass index; ADL, Activities of Daily Living; HAMD, Hamilton Depression Rating Scale; NIHSS, National Institutes of Health Stroke Scale; Hcrt, hypocretin; NfH, neurofilament heavy chain; IL-1β, interleukin-1β. Continuous variables were compared using independent-samples t-tests; categorical variables were compared using Pearson’s chi-squared (χ^2^) test.

In contrast, several variables differed significantly between groups. The proportion of patients aged ≥ 65 years was higher in the sleep disorder group (69.57% vs. 52.63%; χ^2^ = 7.373, *p* = 0.007). A history of snoring was more prevalent among patients with sleep disorders (51.55% vs. 30.53%; χ^2^ = 10.741, *p* = 0.001), as was a history of previous stroke (57.76% vs. 38.95%; χ^2^ = 8.464, *p* = 0.004). Patients in the sleep disorder group demonstrated significantly lower ADL scores at admission (69.64 ± 6.35 vs. 72.22 ± 7.45; *t* = 2.942, *p* = 0.004), higher HAMD scores (8.97 ± 1.62 vs. 6.43 ± 0.57; *t* = 14.743, *p* < 0.001), and higher NIHSS scores (9.48 ± 2.26 vs. 8.75 ± 1.48; *t* = 2.811, *p* = 0.005) at admission (Table 1).

### 3.2 Serum biomarker levels

Serum biomarker concentrations differed markedly between the two groups (Table 1; Figure 1; Supplementary Figure S1). Patients in the sleep disorder group exhibited significantly lower serum Hcrt levels (21.67 ± 4.28 vs. 27.88 ± 6.26 μg/L; *t* = 9.406, *p* < 0.001). Conversely, serum NfH levels were substantially elevated in the sleep disorder group (74.65 ± 21.63 vs. 53.72 ± 10.76 ng/L; *t* = 8.805, *p* < 0.001), as were serum IL-1β levels (62.75 ± 10.33 vs. 52.34 ± 8.57 pg/mL; *t* = 8.282, *p* < 0.001). The effect sizes for between-group biomarker differences, expressed as Cohen’s d, were 1.30 for Hcrt, 1.23 for NfH, and 1.08 for IL-1β, indicating large and clinically meaningful group separations.

### 3.3 Multivariate logistic regression analysis

Multivariate binary logistic regression was performed incorporating all nine variables that achieved significance in the univariate analysis. Multicollinearity diagnostics confirmed that all variance inflation factors were below 10. The variable assignment scheme is presented in Table 2. The Hosmer-Lemeshow test indicated satisfactory model calibration (*p* > 0.05).

**Table 2:** Variable assignment for multivariate logistic regression analysis.

Independent variable	Assignment
Age	< 65 years = 0; ≥ 65 years = 1
History of snoring	No = 0; Yes = 1
Previous stroke history	No = 0; Yes = 1
ADL score at admission	Continuous variable, original value
HAMD score at admission	Continuous variable, original value
NIHSS score at admission	Continuous variable, original value
Serum Hcrt	Continuous variable, original value
Serum NfH	Continuous variable, original value
Serum IL-1β	Continuous variable, original value

**Figure 3:**
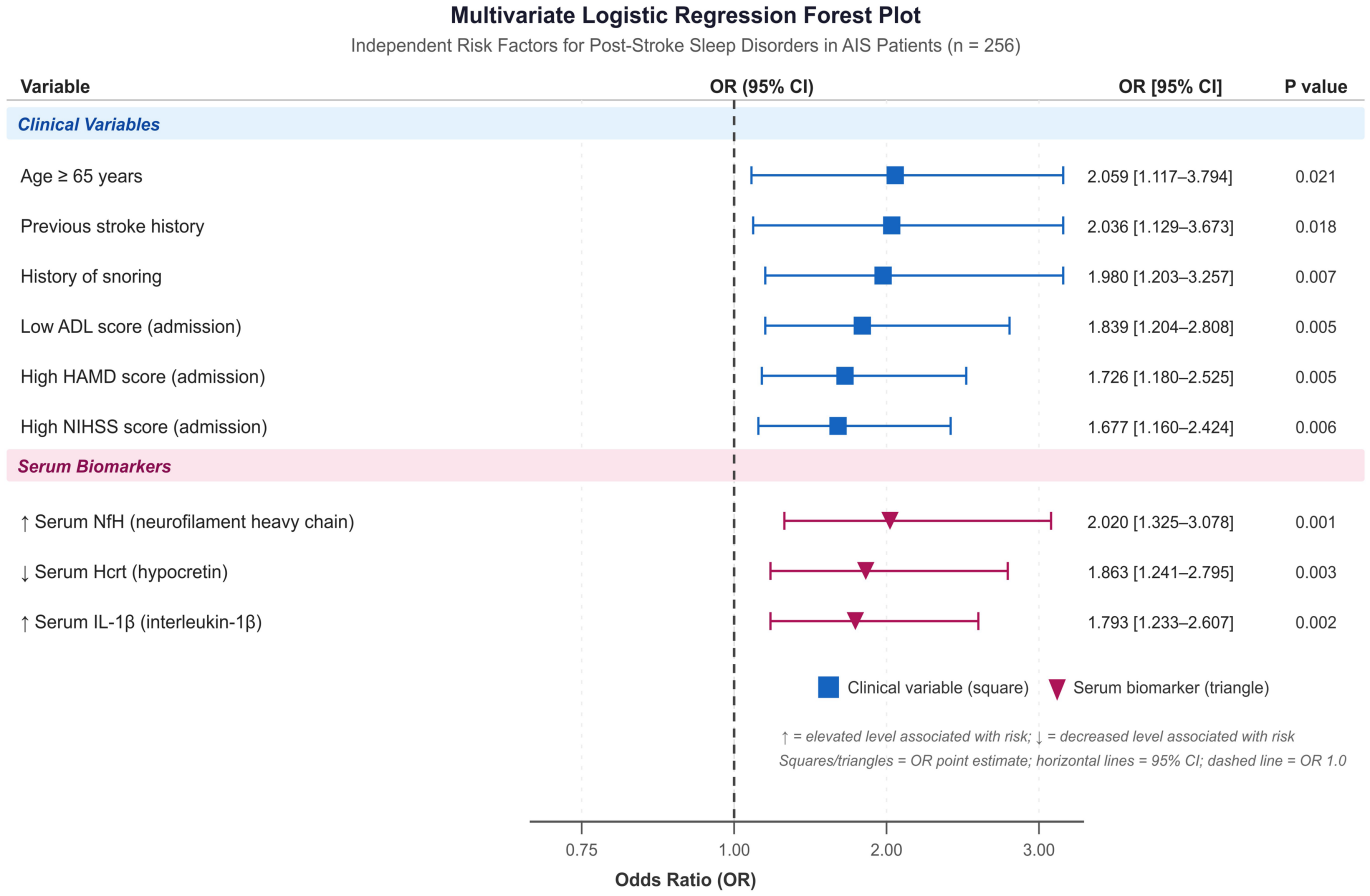
Forest plot of odds ratios (ORs) with 95% confidence intervals (CIs) derived from multivariate logistic regression analysis of independent risk factors for post-stroke sleep disorders. Each square represents the OR point estimate; horizontal lines represent the 95% CI. The vertical dashed reference line is placed at OR = 1.0. Clinical variables are shown in blue; serum biomarkers are shown in red with directional arrows (↓ decrease, ↑ increase) indicating the direction of association with risk. AIS, acute ischemic stroke; ADL, Activities of Daily Living; HAMD, Hamilton Depression Rating Scale; NIHSS, National Institutes of Health Stroke Scale; Hcrt, hypocretin; NfH, neurofilament heavy chain; IL-1β, interleukin-1β.

As shown in Table 3 and Figure 3, all nine variables remained statistically significant in the multivariate model. Age ≥ 65 years was associated with approximately twice the odds of developing sleep disorders (OR = 2.059, 95% CI: 1.117–3.794; *p* = 0.021). A history of snoring (OR = 1.980, 95% CI: 1.203–3.257; *p* = 0.007) and previous stroke history (OR = 2.036, 95% CI: 1.129–3.673; *p* = 0.018) also emerged as significant independent predictors. Lower ADL scores at admission were independently associated with increased risk (OR = 1.839, 95% CI: 1.204–2.808; *p* = 0.005), as were higher HAMD scores (OR = 1.726, 95% CI: 1.180–2.525; *p* = 0.005) and higher NIHSS scores (OR = 1.677, 95% CI: 1.160–2.424; *p* = 0.006).

**Table 3:** Multivariate logistic regression analysis of risk factors for sleep disorders in AIS patients.

Variable	β	SE	Wald χ^2^	*P* value	OR	95% CI
Age ≥ 65 years	0.722	0.312	5.355	0.021	2.059	1.117–3.794
History of snoring	0.683	0.254	7.231	0.007	1.980	1.203–3.257
Previous stroke history	0.711	0.301	5.580	0.018	2.036	1.129–3.673
Low ADL score at admission	0.609	0.216	7.949	0.005	1.839	1.204–2.808
High HAMD score at admission	0.546	0.194	7.921	0.005	1.726	1.180–2.525
High NIHSS score at admission	0.517	0.188	7.563	0.006	1.677	1.160–2.424
Low serum Hcrt	0.622	0.207	9.029	0.003	1.863	1.241–2.795
High serum NfH	0.703	0.215	10.691	0.001	2.020	1.325–3.078
High serum IL-1β	0.584	0.191	9.349	0.002	1.793	1.233–2.607

β, regression coefficient; SE, standard error; OR, odds ratio; CI, confidence interval; AIS, acute ischemic stroke; ADL, Activities of Daily Living; HAMD, Hamilton Depression Rating Scale; NIHSS, National Institutes of Health Stroke Scale; Hcrt, hypocretin; NfH, neurofilament heavy chain; IL-1β, interleukin-1β. Results are derived from multivariate binary logistic regression; p values correspond to the Wald χ^2^ test.

All three serum biomarkers independently contributed to prediction. Lower serum Hcrt levels were associated with elevated risk (OR = 1.863, 95% CI: 1.241–2.795; *p* = 0.003), while higher serum NfH demonstrated the strongest association among the biomarkers (OR = 2.020, 95% CI: 1.325–3.078; *p* = 0.001). Elevated serum IL-1β levels were also independently predictive (OR = 1.793, 95% CI: 1.233–2.607; *p* = 0.002). These results are detailed in Table 3.

The corresponding nomogram is presented in Figure 2. On the point scale, NfH carries the widest axis (0–10 pts., range 30–105 ng/L), reflecting its largest β × range product among all predictors. The continuous clinical and biomarker predictors (ADL, Hcrt, IL-1β, HAMD, and NIHSS) each contribute intermediate ranges of 7.35–8.85 pts., while the three binary predictors (age ≥ 65 years, snoring history, and prior stroke history) contribute approximately 2.4–2.6 pts. each. Optimal biomarker cutoff values from ROC analysis are marked on their respective axes. The Total Points axis spans 0–58; a predicted probability of ≥ 0.90 corresponds to a total score of approximately 35 points or above.

### 3.4 Predictive performance of serum biomarkers: ROC curve analysis

**Figure 4:**
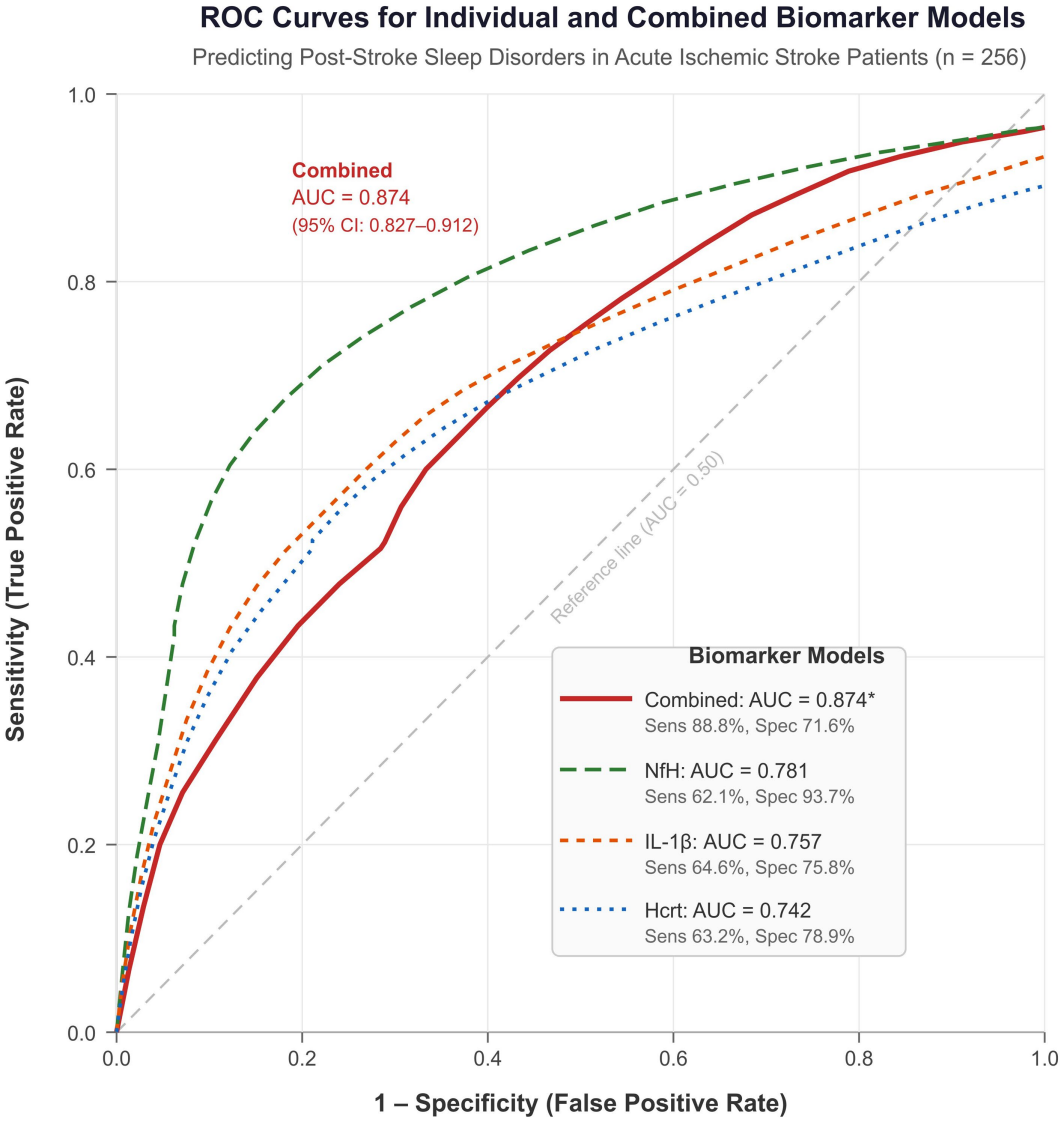
Receiver operating characteristic (ROC) curves for serum Hcrt, NfH, IL-1β, and their combination in predicting sleep disorders in patients with AIS. The solid red line represents the combined detection model; dashed lines represent individual biomarkers. The diagonal reference line represents no discriminative ability (AUC = 0.50). AUC values and 95% CIs are displayed in the figure legend. ^*^The combined model AUC was significantly superior to each individual biomarker (DeLong test, all *p* < 0.05). Optimal cutoffs: Hcrt ≤ 25.42 μg/L; NfH ≥ 67.41 ng/L; IL-1β ≥ 59.41 pg/mL (Youden index). ROC curves are smoothed schematic representations based on reported AUC, sensitivity, and specificity values. AIS, acute ischemic stroke; Hcrt, hypocretin; NfH, neurofilament heavy chain; IL-1β, interleukin-1β; AUC, area under the curve.

ROC curve analysis was performed to evaluate the discriminative capacity of serum Hcrt, NfH, and IL-1β levels for predicting sleep disorders in AIS patients (Table 4; Figure 4). Serum Hcrt yielded an AUC of 0.742 (95% CI: 0.684–0.794; *p* < 0.001) at an optimal cutoff of 25.42 μg/L, with a sensitivity of 63.16% and specificity of 78.88%. Serum NfH demonstrated an AUC of 0.781 (95% CI: 0.725–0.830; *p* < 0.001) at a cutoff of 67.41 ng/L, with a sensitivity of 62.11% and specificity of 93.68%. Serum IL-1β showed an AUC of 0.757 (95% CI: 0.699–0.808; *p* < 0.001) at a cutoff of 59.41 pg/mL, with a sensitivity of 64.60% and specificity of 75.79%.

**Table 4:** Predictive value of serum Hcrt, NfH, and IL-1β levels for sleep disorders in AIS patients by ROC analysis.

Biomarker	Cutoff value	AUC	95% CI	*P* value	Sensitivity (%)	Specificity (%)
Hcrt	25.42 μg/L	0.742^a^	0.684–0.794	<0.001	63.16	78.88
NfH	67.41 ng/L	0.781^a^	0.725–0.830	<0.001	62.11	93.68
IL-1β	59.41 pg/mL	0.757^a^	0.699–0.808	<0.001	64.60	75.79
Combined^a^	—	0.874	0.827–0.912	<0.001	88.82	71.58

AUC, area under the curve; CI, confidence interval; Hcrt, hypocretin; NfH, neurofilament heavy chain; IL-1β, interleukin-1β. AUC values were computed from ROC curve analysis; optimal cutoffs determined using the Youden index. Pairwise AUC comparisons performed using the DeLong test.

a *P* < 0.05 compared with the combined model (DeLong test).

The combined model integrating all three biomarkers achieved a superior AUC of 0.874 (95% CI: 0.827–0.912; *p* < 0.001), with a sensitivity of 88.82% and specificity of 71.58%. DeLong test comparisons confirmed that the AUC of the combined model was significantly higher than that of each individual biomarker (all *p* < 0.05; Table 4; Figure 4).

### 3.5 Internal validation

Bootstrap resampling validation (1,000 iterations) of the combined three-biomarker logistic regression model yielded a mean optimism-corrected AUC of 0.861 (95% bootstrap confidence interval: 0.812–0.903). The optimism was estimated at 0.013, indicating that the apparent AUC of 0.874 was minimally inflated and that the model’s discriminative performance is robust with negligible evidence of overfitting. The calibration plot (Supplementary Figure S2) demonstrated good concordance between predicted probabilities and observed event proportions across deciles of predicted risk, with data points closely approximating the 45-degree line of perfect calibration (mean absolute error = 0.028), further supporting the reliability of the model.

## 4 Discussion

AIS is a common neurological condition in which acute cerebrovascular occlusion leads to focal ischemic brain injury, typically manifesting as sudden-onset hemiplegia, aphasia, and other neurological deficits (Kumar et al., 2025). In addition to motor and cognitive sequelae, sleep disorders are increasingly recognized as frequent non-motor complications after AIS, with implications not only for rehabilitation and quality of life but also for the risk of stroke recurrence, depression, and cognitive decline (Geng et al., 2023; Li et al., 2023). The pathogenesis of post-stroke sleep disturbances is complex, involving neuroanatomical damage, neurotransmitter imbalance, inflammatory activation, and psychological stress. Accordingly, the identification of risk factors and relevant biomarkers is of considerable clinical significance for the early detection of high-risk patients and the implementation of targeted interventions.

The present study identified six clinical risk factors that were independently associated with sleep disorders in patients with AIS: age ≥ 65 years, history of snoring, previous stroke history, low ADL score at admission, and high HAMD and NIHSS scores at admission.

The association between advanced age and post-stroke sleep disturbance is consistent with the established understanding that aging is accompanied by physiological changes in sleep architecture, including reductions in slow-wave sleep, as well as an increased burden of comorbidities and degenerative changes in brainstem sleep-regulatory centers that collectively heighten vulnerability to sleep disruption (Chen et al., 2024; Zhang Y. et al., 2023). The finding that a history of snoring was an independent risk factor is noteworthy, as habitual snoring may indicate the presence of subclinical or undiagnosed obstructive sleep apnea. In the post-stroke setting, such patients may be particularly susceptible to nocturnal hypoventilation and sleep fragmentation due to exacerbated upper airway obstruction resulting from inflammatory edema and reduced upper airway muscle tone (Lau et al., 2019; Liu et al., 2026).

Patients with a history of prior stroke may harbor residual structural or functional damage to neural networks involved in sleep–wake regulation, including thalamic and brainstem circuits. This pre-existing vulnerability could amplify the risk of sleep disturbance following a subsequent ischemic event (Hasan et al., 2021). The independent association of lower ADL scores with sleep disorders is plausible, as reduced functional independence often limits daytime physical activity and light exposure, which in turn may disrupt circadian rhythm entrainment (Cai et al., 2025; Sherrill et al., 1998). The role of depressive symptoms, reflected by elevated HAMD scores, aligns with the well-documented bidirectional relationship between depression and sleep disturbance; patients with depressive features often exhibit altered sleep architecture, including early morning awakening and poor sleep continuity (Nutt et al., 2008). Finally, the association of higher NIHSS scores with sleep disorders likely reflects the fact that more severe neurological impairment may indicate greater involvement of sleep–wake regulatory pathways, thereby increasing the likelihood of sleep disruption (Lucke-Wold et al., 2015; Shen et al., 2023).

Beyond the clinical variables, the present study demonstrated that decreased serum Hcrt and elevated serum NfH and IL-1β levels were each independently associated with post-stroke sleep disorders, highlighting the role of neurobiochemical and inflammatory mechanisms.

Hcrt is a key neuropeptide involved in the maintenance of wakefulness and regulation of the sleep–wake cycle. The observed decrease in serum Hcrt levels in patients with sleep disorders may reflect ischemia-induced damage to hypothalamic neurons, leading to circadian rhythm dysregulation and insufficient wakefulness drive, which can manifest as excessive daytime sleepiness and nocturnal sleep fragmentation (Chen et al., 2024; Rissardo et al., 2025). The optimal serum Hcrt cutoff of 25.42 μg/L identified here may serve as a candidate threshold for clinical screening, subject to external validation across patient populations and assay platforms. NfH is a well-established biomarker of neuronal axonal injury. The elevated NfH levels observed in the sleep disorder group suggest that more severe axonal damage after AIS may impair the neural conduction processes governing sleep–wake transitions. This damage potentially involves brainstem, thalamic, and cortical neural pathways critical for sleep–wake regulation. However, it is important to acknowledge that NfH is a marker of general neuroaxonal injury and does not provide anatomical specificity regarding which neural pathways are affected. The observed elevation in the sleep disorder group may therefore partially reflect the overall severity of ischemic brain injury rather than selective damage to sleep-regulatory circuits. Accordingly, the relationship between NfH and post-stroke sleep disturbance should be interpreted as associative rather than mechanism-specific. This interpretation is consistent with findings from Zhang et al. (2021). Additionally, our findings are consistent with the broader neurofilament literature demonstrating that circulating neurofilament proteins correlate with structural brain injury and functional outcomes across neurological conditions (Shahim et al., 2024; Sanchez et al., 2025). IL-1β is a potent proinflammatory cytokine released in substantial quantities following cerebral ischemia. Elevated IL-1β levels are indicative of an active inflammatory response, which may affect hypothalamic function, alter neurotransmitter metabolism, and disrupt normal sleep architecture, particularly slow-wave sleep, thereby increasing the risk of sleep disorders (Zhang N. et al., 2025).

Importantly, serum IL-1β is a pleiotropic cytokine produced by a wide array of cell types throughout the body, including peripheral monocytes, macrophages, and endothelial cells, and is not exclusively of central nervous system origin. Elevated serum levels in the post-stroke setting may therefore reflect systemic inflammatory processes—including the general acute-phase response and potential subclinical infections—rather than inflammation specifically confined to the brain. The observed association between IL-1β and sleep disturbance is thus best regarded as an indirect, associative relationship, and the contribution of systemic versus central inflammatory sources cannot be disentangled on the basis of serum measurements alone (Lopez-Castejon and Brough, 2011).

Although accumulating evidence supports the individual contributions of these biomarkers to neurological outcomes after AIS, studies that simultaneously examine neuroendocrine dysregulation, axonal injury, and inflammation as combined predictors of post-stroke sleep disorders have been limited. Emerging research has explored alternative predictive approaches: a gut microbiota profiling model achieved an AUC of 0.768 for post-stroke sleep disorder prediction (Xie et al., 2023), and a clinical-psychological decision tree model incorporating anxiety, social support, fatigue, and stroke severity achieved high internal validation accuracy (Sun X. et al., 2025). However, neither approach simultaneously targets the three pathophysiological axes addressed in the present study. The combined assessment of serum Hcrt, NfH, and IL-1β provided substantially greater predictive accuracy for post-stroke sleep disorders (AUC = 0.874) than any single biomarker alone. Bootstrap internal validation demonstrated that this discriminative performance was robust, with a mean optimism-corrected AUC of 0.861 and negligible optimism (0.013), mitigating concerns of overfitting to the derivation sample. This observation suggests that the three biomarkers capture distinct but complementary dimensions of the pathophysiology underlying post-stroke sleep disruption: neuroendocrine dysregulation (Hcrt), structural axonal damage (NfH), and inflammation (IL-1β). Compared with previous studies that focused on single-mechanism indicators, such as the relationship between Hcrt and sleep fragmentation (Fu et al., 2024), the present study is among the first to simultaneously evaluate these three biomarker categories, offering a more integrated perspective on the multidimensional pathophysiology of sleep disorders. Nevertheless, the observed associations should be interpreted with appropriate caution, as these biomarkers are indirect indicators of broad pathophysiological processes; the between-group differences may partly reflect overall stroke severity or systemic inflammatory burden rather than specific disruption of sleep–wake circuits.

The clinical utility of the combined model is further supported by its high sensitivity (88.82%), which minimizes the probability of failing to identify high-risk patients—a critical property for a screening tool intended for routine use. The trade-off of moderate specificity (71.58%) may produce some over-referral for sleep evaluation; however, given the serious prognostic implications of post-stroke sleep disorders, a high-sensitivity screening approach is clinically appropriate. To facilitate bedside implementation, a nomogram was constructed from the multivariate model coefficients (Figure 2), enabling individualized risk estimation from the nine identified predictors without requiring statistical software. Such visual risk calculators have demonstrated practical value in analogous post-stroke complication prediction contexts (Sun X. et al., 2025).

Several limitations merit acknowledgement. First, the single-institution recruitment design carries inherent risks of selection and referral bias, as the patient demographics, clinical management protocols, and referral patterns at our center may not be representative of broader populations. The geographic and ethnic homogeneity of the cohort further limits the extent to which these findings can be extrapolated to populations of differing demographic composition. Prospective, multicenter validation studies involving geographically and ethnically diverse populations are essential to confirm the generalizability of the proposed biomarker panel and the predictive model before clinical adoption. Second, the PSQI assesses global sleep quality and does not capture specific sleep disorder subtypes or objective sleep parameters. In the acute post-stroke setting, cognitive impairment, aphasia, and the unfamiliar hospital environment may further compromise the reliability of self-reported sleep quality. The absence of polysomnographic or actigraphic validation means that physiologically defined sleep disturbances—including obstructive sleep apnea, periodic limb movement disorder, and REM sleep behavior disorder—may have been underdiagnosed or misclassified. Such outcome misclassification could attenuate true associations, introduce noise into model parameter estimates, and artificially inflate or deflate apparent predictive performance. Future investigations should incorporate objective sleep monitoring alongside subjective instruments to improve diagnostic precision. Third, the cross-sectional biomarker assessment precludes causal inference. Fourth, although bootstrap internal validation yielded reassuring results (optimism-corrected AUC = 0.861), this does not substitute for external validation in independent cohorts. Fifth, the lesion-location classification employed in this study was necessarily broad, categorizing infarcts into large anatomical regions (basal ganglia, cerebral cortex, brainstem, cerebellum). Key structures governing sleep–wake regulation—including the hypothalamic suprachiasmatic nucleus and ventrolateral preoptic nucleus, thalamic reticular and relay nuclei, and brainstem arousal centers such as the locus coeruleus and pedunculopontine tegmental nucleus—are small subcortical structures that would be subsumed within these broad categories. Consequently, the non-significant finding for lesion location should not be interpreted as evidence against a neuroanatomical contribution to post-stroke sleep disturbance, but rather as a reflection of insufficient spatial resolution in the classification scheme. Future studies should employ high-resolution structural MRI with atlas-based or voxel-level lesion mapping to more precisely delineate the relationship between stroke topography and sleep outcomes (Krick et al., 2023). Sixth, C-reactive protein (CRP), procalcitonin, and other established clinical markers of systemic infection were not systematically quantified at the time of serum biomarker measurement. Given that hospitalized stroke patients are at well-documented risk for nosocomial infections—including aspiration pneumonia and urinary tract infections—which could independently elevate circulating IL-1β levels, the absence of concurrent systemic inflammatory markers represents a potential confounding factor that should be addressed in future studies. Future multicenter, prospective studies with objective sleep monitoring and longitudinal biomarker assessments are warranted to validate these findings.

## 5 Conclusion

In conclusion, sleep disorders in patients with AIS are independently associated with age ≥ 65 years, snoring history, prior stroke history, lower ADL scores, and higher HAMD and NIHSS scores at admission, as well as with decreased serum Hcrt and elevated serum NfH and IL-1β levels. The combined assessment of serum Hcrt, NfH, and IL-1β provides substantially superior predictive performance compared with individual biomarkers (AUC 0.874 vs. 0.742–0.781; bootstrap-corrected AUC 0.861), supporting the clinical utility of a multi-biomarker approach targeting three distinct pathophysiological axes. A nomogram derived from the multivariate model offers a practical tool for individualized risk stratification. These biomarkers should be regarded as indirect, associative indicators of broad pathophysiological processes, and the observed predictive relationships require confirmation through prospective multicenter validation and formal nomogram calibration before implementation in routine clinical practice.

## Data Availability

The raw data supporting the conclusions of this article will be made available by the authors, without undue reservation.

## Supplementary material

SUPPLEMENTARY FIGURE S1 Grouped box plots illustrating the distribution of serum Hcrt (μg/L), NfH (ng/L), and IL-1β (pg/mL) levels in the sleep disorder (*n* = 161) and non-sleep disorder (*n* = 95) groups. Dashed reference lines indicate optimal ROC-derived cutoff values. All between-group comparisons *P* < 0.001.

SUPPLEMENTARY FIGURE S2 Calibration plot for the combined three-biomarker model. Observed event proportions are plotted against predicted probabilities across deciles of predicted risk. The diagonal dashed line represents perfect calibration (mean absolute error = 0.028; Hosmer–Lemeshow *P* > 0.05).
