# Supplementary figures and images for: Serum hypocretin, neurofilament heavy chain, and interleukin-1β as combined predictors of sleep disorders following acute ischemic stroke
Source: Front Neurosci. 2026 May 7;20:1814307. doi: 10.3389/fnins.2026.1814307 (PMC13220776; doi:10.3389/fnins.2026.1814307)

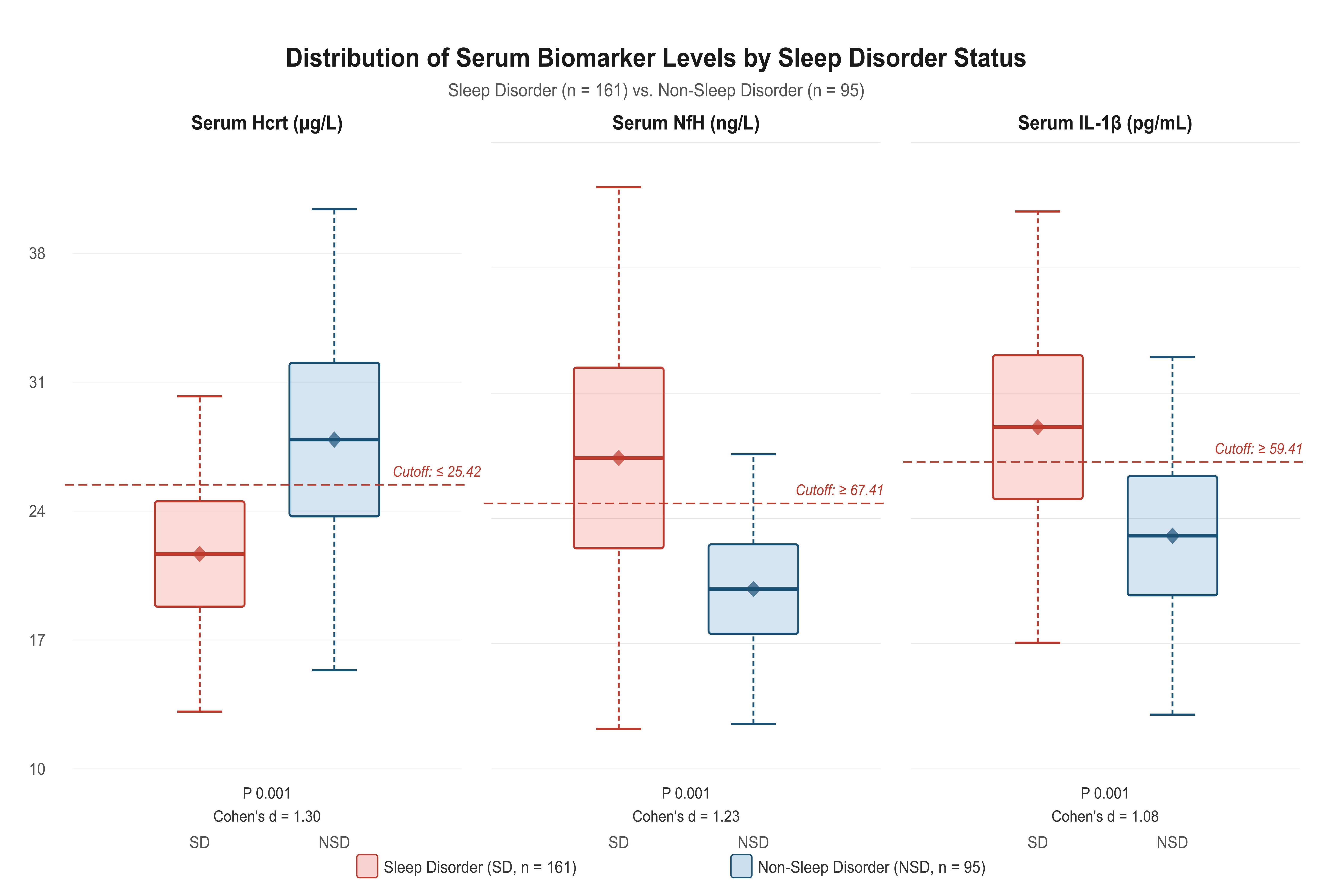

Supplement: SUPPLEMENTARY FIGURE S1 — Grouped box plots illustrating the distribution of serum Hcrt (μg/L), NfH (ng/L), and IL-1β (pg/mL) levels in the sleep disorder (n = 161) and non-sleep disorder (n = 95) groups. Dashed reference lines indicate optimal ROC-derived cutoff values. All between-group comparisons P < 0.001. [file Image_1.jpeg]

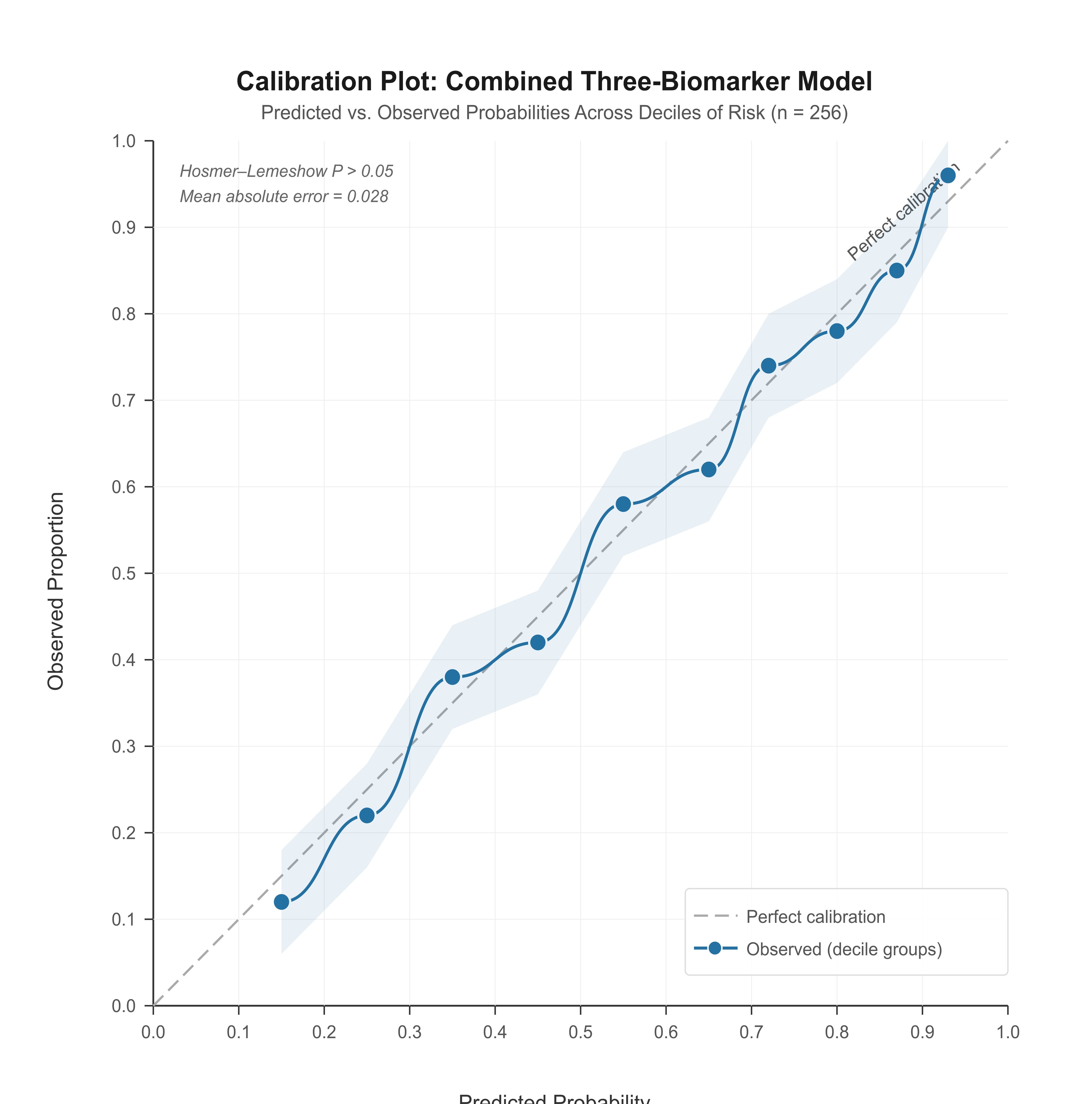

Supplement: SUPPLEMENTARY FIGURE S2 — Calibration plot for the combined three-biomarker model. Observed event proportions are plotted against predicted probabilities across deciles of predicted risk. The diagonal dashed line represents perfect calibration (mean absolute error = 0.028; Hosmer–Lemeshow P > 0.05). [file Image_2.jpeg]
